# Simultaneous Imaging of Two Different Cancer Biomarkers Using Aptamer-Conjugated Quantum Dots

**DOI:** 10.3390/s150408595

**Published:** 2015-04-13

**Authors:** Jonghwan Lee, Hyo Jin Kang, Hyeok Jang, Youn Jung Lee, Yong Seung Lee, Bahy A. Ali, Abdulaziz A. Al-Khedhairy, Soonhag Kim

**Affiliations:** 1 Institute for Bio-Medical Convergence, College of Medicine, Catholic Kwandong University, Gangneung-si, Gangwon-do, 270-701, Korea; E-Mails: jonghwanlee104@gmail.com (J.L.); hyojinkang.bio@gmail.com (H.J.K.); zzzang1265@naver.com (H.J.); hotdog0903@naver.com (Y.J.L.); inyasio0731@naver.com (Y.S.L.); 2 Catholic Kwandong University International St. Mary’s Hospital, Incheon Metropolitan City, 404-834, Korea; 3 Al-Jeraisy, Chair for DNA Research, Department of Zoology, College of Science, King Saud University, Riyadh 11451, Saudi Arabia; E-Mails: bahali@ksu.edu.sa (B.A.A.); kedhairy@ksu.edu.sa (A.A.A.-K.); 4 Department of Nucleic Acids Research, Genetic Engineering and Biotechnology Research Institute, City for Scientific Research and Technological Applications, Alexandria 21934, Egypt

**Keywords:** quantum dot, aptamer, tenascin-C, nucleolin

## Abstract

Studying gene expression profile in a single cancer cell is important because multiple genes are associated with cancer development. Quantum dots (QDs) have been utilized as biological probes for imaging and detection. QDs display specific optical and electrical properties that depend on their size that can be applied for imaging and sensing applications. In this study, simultaneous imaging of the cancer biomarkers, tenascin-C and nucleolin, was performed using two types of aptamer-conjugated QDs. The simultaneous imaging of these two different cancer markers in three cancer cell lines was reliable and cell line-specific. Current requirements for cancer imaging technologies include the need for simple preparation methods and the ability to detect multiple cancer biomarkers and evaluate their intracellular localizations. The method employed in this study is a feasible solution to these requirements.

## 1. Introduction

The number of genes in well-characterized genomes of multicellular eukaryotes ranges from thousands to hundreds of thousands, and hundreds of genes are required to sustain a functioning cell under single environmental condition. High throughput analysis, including expressed sequence tag (EST), DNA microarray, subtractive cloning, differential display, and serial analysis of gene expression (SAGE), has become a well-established tool set for the parallel monitoring of gene expression profiles in a single cell. However, none of these can provide information on gene expression in intact cells. Bioimaging approaches provide diagnostic applications, revealing biological and functional roles of genes in intact cells. Most of these studies, especially in cancer, have been limited to single genes [1,2].Bioimaging of multiple genes in a single cell has become possible due to the recent development of nanotechnology, including quantum dots (QDs) and surface chemistry.

QDs are nano-scaled light-emitting particles that have the potential for diagnostic and therapeutic applications in the fields of bio-imaging due to their many unique optical properties, including broad absorption with narrow photoluminescence spectra, high quantum yield, low photobleaching, and resistance to chemical degradation. The simultaneous excitation of multiple QDs emitting unique wavelengths is applicable for multiplexed bioimaging. Surface-modified QDs conjugated with small oligonucleotide ligands (aptamers), peptides, or small molecules bound to antigens present on target cells have been used to study targeted imaging, therapy, and drug delivery in cancer [3,4,5].

In previous studies, we developed a new method for simultaneously imaging two cancer biomarkers in a single cancer cell line using two different QDs conjugated with an aptamer or a peptide [6]. The two cancer biomarkers were integrin α_v_β_3_, which is comprised of heterodimeric transmembrane cell adhesion molecules, and integrin, which binds to arginine-glycine-aspartic acid (RGD)-containing components of the interstitial matrix, such as vitronectin, fibronectin, and thrombospondin (which plays a key role in tumor angiogenesis and metastasis [7,8]), and nucleolin, which is known to be a nuclear protein in tumors, and is expressed on the surface of endothelial cells during angiogenesis [9] and is involved in the regulation of cell proliferation, cytokinesis, replication, embryogenesis, and nucleogenesis [10,11].

In this study, we conducted simultaneous cancer biomarker imaging in three different cancer cell lines using two different QDs, QD605 and AD655, which are conjugated to tenascin-C aptamer (TTA-1) and AS1411 aptamer, respectively. TTA-1 aptamer binds specifically to tenascin-C, an extracellular matrix protein overexpressed in cancer cells [12,13]. AS1411 is an aptamer that targets nucleolin in the plasma membranes of cancer cells [14]. QD-aptamer conjugates were simultaneously administered to DU145 cells (a human prostate cancer cell line), U-87 MG cells (a human glioblastoma-astrocytoma cell line), and A549 cells (a human lung carcinoma cell line), and simultaneous fluorescence imaging was confirmed with a confocal laser microscope.

## 2. Experimental Section

### 2.1. Preparation of QD-Aptamer Conjugates

Two carboxyl-terminated QDs with different wavelengths, QD605 (605 nm, Invitrogen, Carlsbad, CA, USA) and QD655 (655 nm, Invitrogen), were conjugated with two amine-terminated aptamers: TTA-1 aptamer (H_2_N-C6-5’ CCTGCACTTGGCTTGGATTTCAGAAGGGAGACCC-3’-OH, Postech Biotech Center) and AS1411 aptamer (H_2_N-C6-5’ TTGGTGGTGGTGGTTGTGGTGGTGGTGG-3’-OH, Postech Biotech Center), respectively. As a negative control aptamer, an amine-terminated oligonucleotide was synthesized (H_2_N-C6-5’-TTCCTCCTCCTCCTTCTCCTCCTCCTCC-3’-OH, BIONICS, Seoul, Korea). 10 pmol of QDs were conjugated with 400 pmol of aptamers at a molar ratio of 1:40 in 10 mM Tris buffer (pH 7.4) using a coupling reagent N-(3-dimethylaminopropyl)-N’-ethyl-carbodiimide hydrochloride (EDC, 40 nmol). The mixture was vigorously vortexed and incubated for 1 h at room temperature. Unconjugated aptamers were removed, and conjugated QD-aptamers were recovered by centrifugal filtration at 13,000 rpm for 30 min. Followed by washing with Tris buffer (pH 7.4), the aggregated mixtures were dispersed by brief sonication (22 kHz, amplitude 12 μm, with a sonication time of 120 s) using an Ultrasonic Processor (SONICS & MATERIALS, INC., Newtown, CT, USA).

### 2.2. Characterization of QD-Aptamer Conjugates

To analyze the prepared QD-aptamer conjugates, transmission electron microscopy (TEM, JEM 1010, JEOL, Japan) was performed. Size differences were analyzed with ImageJ software and by electrophoretic mobility shift assay. QD-aptamer conjugates, unconjugated QDs, and QD-mutant aptamer conjugates were loaded on a 2% agarose gel. The conjugation efficiency was evaluated by measuring the concentration of unconjugated aptamers in supernatants collected by centrifugal filtration.

### 2.3. Cell Culture Conditions

DU145 cells, U-87 MG cells, and A549 cells were grown in Dulbecco’s Modified Eagle medium (DMEM, Thermo Scientific Hyclone, Waltham, MA, USA) supplemented with 10% fetal bovine serum (FBS, Gibco, Grand Island, NY, USA) and 1% penicillin/streptomycin (Gibco). CHO cells were grown in Ham’s F-12 medium (Sigma-Aldrich, St. Louis, MO, USA).

### 2.4. Treatment with QD-Aptamer Conjugates and Measurement of Fluorescence Intensity

Cells (1 × 10^5^) were seeded into 24-well culture plates. After 24 h, cells were cultured at 4 °C for 30 min to minimize nonspecific binding. Cells were washed with PBS and culture medium was replaced with Tris buffer (pH 7.4). QD-aptamer conjugates were then introduced to cells and cultured for 30 min at 37 °C. Followed by washing three times with PBS buffer, cells were trypsinized using 0.25% trypsin/EDTA (Gibco). To measure fluorescence intensity, cells (100 μL) were collected in Tris buffer and transferred to 96-well assay plates (Chemicall GmbH, Germany). Fluorescence intensity was measured using a Cary Eclipse fluorescence spectrophotometer (Varian, Inc., Walnut Creek, CA, USA) and presented as means ± standard deviation (SD) of triplicate samples (* *P* < 0.05, ** *P* < 0.005).

### 2.5. Reverse Transcription-Polymerase Chain Reaction (RT-PCR)

Total RNA was purified from cells using RNeasy Plus Mini Kit (Qiagen, Valencia, CA, USA) and cDNA was prepared using QuantiTect Reverse Transcription Kit (Qiagen). Following primers were used to amplify genes: tenascin-C, forward (5’-GTGAAGGCATCCACTGAACAAGC-3’) and reverse (5’-TTGTGCTGAAGTCCTGAGTGACC-3’); nucleolin, forward (5’-AATGAGGGCAGAGCAATCAGG-3’) and reverse (5’-GTCAGTAACTATCCTTGCCCC-3’); beta-actin, forward (5’-CATGTACGTTGCTATCCAGGC-3’) and reverse (5’-CTCCTTAATGTCACGCACGAT-3’).

### 2.6. Confocal Microscopy

Cells (1 × 10^5^) were seeded into 24-well culture plates containing poly-l-lysine-coated coverslips and cultured for 24 h. Before introducing QD-aptamer conjugates, cells were cultured at 4 °C for 30 min to minimize nonspecific binding. Unbound QD-aptamer conjugates were removed by washing three times with PBS buffer. After fixation with 3.7% formaldehyde solution (Sigma-Aldrich), cells were treated with 0.25% Triton X-100 (in PBS) for 15 min and washed with PBS buffer. The coverslips were then mounted onto microscope slides using 4’,6-diamidino-2-phenylindole dihydrochloride (DAPI)-containing VECTASHIELD mounting medium (Vector Laboratories, Inc., CA, USA). Imaging analysis was performed via confocal laser scanning microscopy (LSM 510; Carl Zeiss, Weimer, Germany) using the following filter set; Excitation wavelength: HFT 405/488 nm, and emission wavelength: DAPI imaging: 420–480 nm, QD-TTA-1: 560–615 nm, and QD-AS1411: 625–754 nm.

## 3. Results and Discussion

### 3.1. Characterization of Prepared QD-Aptamer Conjugates

**Figure 1:**
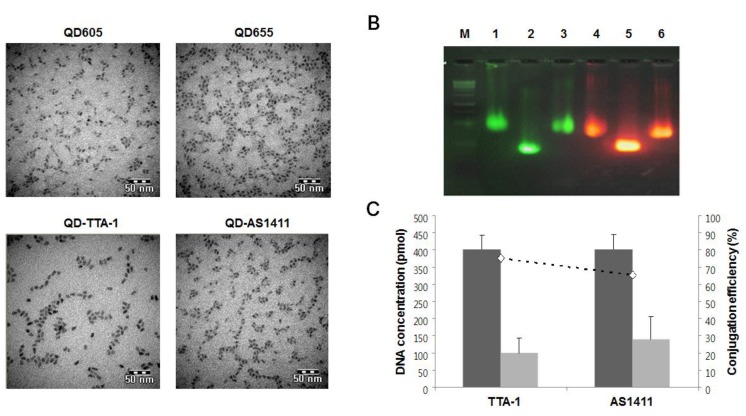
Characterization of quantum dot (QD)-aptamer conjugates. (**A**) TEM images of QDs and QD-aptamer conjugates; (**B**) Gel electrophoresis of QD-TTA-1 and QD-AS1411. QD-TTA-1 (lane 1), QD605 (lane 2), QD-TTA-1mt (lane 3), QD-AS1411 (lane 4), QD655 (lane 5), and QD-AS1411mt (lane 6). Size marker is indicated by “M”. Data evaluated by UV excitation (235/345 nm); (**C**) Conjugation efficiency of QD-TTA-1 and QD-AS1411. Dark gray bars indicate QD-aptamer conjugates and white gray bars indicate unconjugated QDs. Data are displayed as means ± standard deviations of triplicate samples.

Different QDs were conjugated with aptamers at a molar ratio of 1:40 for 1 h at room temperature using 40 nmol of N-(3-dimethylaminopropyl)-N’-ethylcarbodiimide hydrochloride (EDC). After conjugation, mild sonication was applied to the conjugate mixtures to disperse the aggregates. QD605 was conjugated with TTA-1 (QD-TTA-1), and QD655 was conjugated with AS1411 (QD-AS1411). Mutant aptamers (TTA-1mt and AS1411mt) were also conjugated with QDs (designated as QD-TTA-1mt and QD-AS1411mt, respectively). Transmission electron microscopy (TEM) imaging showed that the prepared QD-aptamer conjugates were fully dispersed (Figure 1A). The formation of QD-aptamer conjugates was further confirmed by electrophoretic mobility shift assay using a 2% agarose gel electrophoresis. Aptamer-conjugated QDs showed a distinct mobility shift (Figure 1B). The conjugation efficiency between aptamers and QDs was 75.5% for QD-TTA1 and 65.1% for QD-AS1411 (Figure 1C).

### 3.2. Cancer-Targeting Specificity of QD-Aptamer Conjugates in Three Cancer Cell Lines

**Figure 2:**
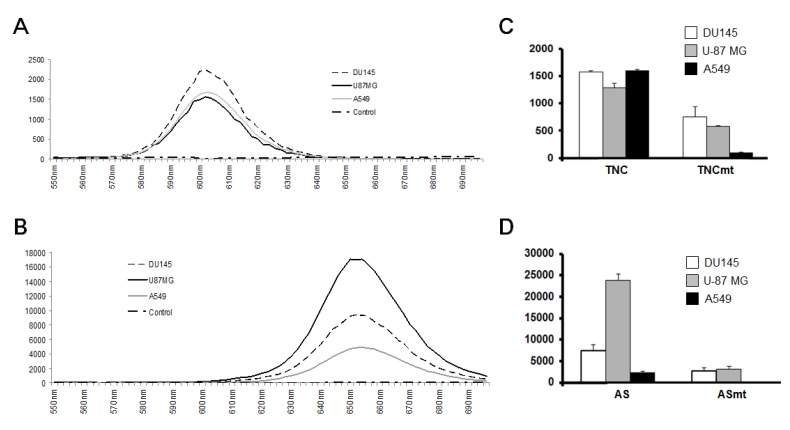
Stability and functionality of QD-aptamer conjugates. Fluorescence emission spectra of (**A**) QD-TTA-1 and (**B**) QD-AS1411 in three cancer cell lines. DU145 (dotted black line), U-87 MG (solid black line), A549 (gray line), and baseline (dash-dotted line); excitation: 480 nm, scanning wavelength: 500–800 nm, with a bandwidth of 10 nm. Fluorescence intensities of (**C**) QD-TTA-1 and QD-TTA-1mt; and (**D**) QD-AS1411 and QD-AS1411mt in three cancer lines. QD-aptamer conjugates were added to cancer cells and the fluorescence intensity was measured. Data are displayed as means ± standard deviations of triplicate samples.

To evaluate the cancer-targeting specificity of each QD-aptamer conjugate, three cancer cell lines, DU145, U-87 MG, and A549 cells, were treated and incubated with each QD-aptamer conjugate. Before QD-aptamer conjugate treatment, 1 × 10^5^ cells were cultured at 4 °C for 30 min to minimize nonspecific binding. Fluorescence emission spectra showed that both QD-aptamer conjugates were stable in all three cancer cell lines. The cellular environment did not result in any peak shift of the QD-aptamer conjugates (Figure 2A,B). The fluorescence intensities were cell line-specific to the three cancer cell lines and distinct from those of the negative control groups (Figure 2C,D). QD-TTA-1 showed a high level of fluorescence intensity in all three cancer cell lines. However, the fluorescence intensity of QD-AS1411 was highest in U-87 MG cells and lowest in A549 cells. These results indicate that there are different expression profiles of tenascin-C and nucleolin in different cancers.

**Figure 3:**
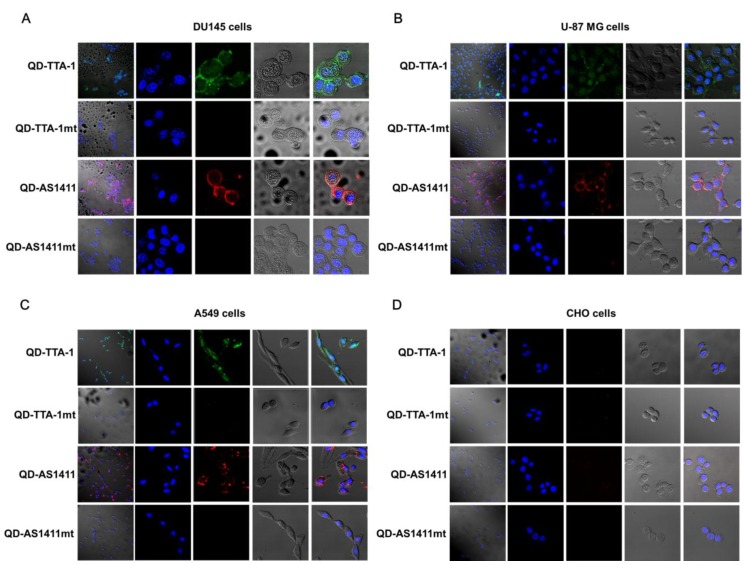
Confocal microscope images of cells incubated with QD-aptamer conjugates. (**A**) DU145; (**B**) U-87 MG; (**C**) A549; and (**D**) CHO cells. The first column shows a magnification of 200X and columns 2 through 5 show a magnification of 800X. Columns 1 and 5: merged fluorescence image of DAPI and bright field and each QD-aptamer conjugate; column 2: DAPI (blue: 420–480 nm); column 3: QD-aptamer conjugates for imaging of tenascin-C (green: 560–615 nm) and imaging of nucleolin (red: 647–690 nm); column 4: bright field.

**Figure 4:**
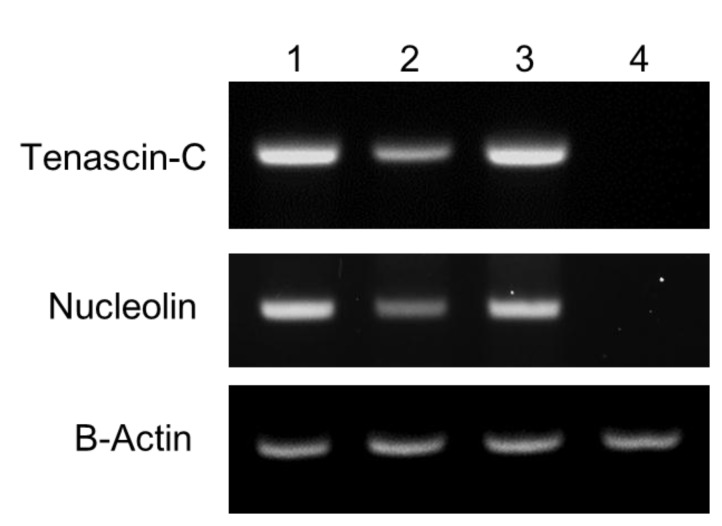
Reverse Transcription-Polymerase Chain Reaction (RT-PCR) analysis of the expression of tenascin-C and nucleolin in four cell lines. Lane 1, DU145 cells; lane 2, U-87 MG cells; lane 3, A549 cells; lane 4, CHO cells.

For confocal microscopy analysis of the target specificity of QD-aptamer conjugates, three cancer cell lines and one normal healthy cell line (Chinese hamster ovary, CHO) were incubated with each QD-aptamer conjugate. QD-TTA-1 and QD-AS1411 showed strong fluorescence signals in both DU145 and A549 cells, and showed slightly weak fluorescence signals in A549 cells (Figure 3A–C). However, CHO cells did not display any fluorescence signal (Figure 3D). The negative controls also displayed no fluorescence signals in any of the four cell lines. Cell line specific expression profiles of tenascin-C and nucleolin in four cell lines were further confirmed by RT-PCR (Figure 4).

### 3.3. Simultaneous Imaging of Two Cancer Biomarkers Using QD-Aptamer Conjugates in Three Cancer Call Lines

**Figure 5:**
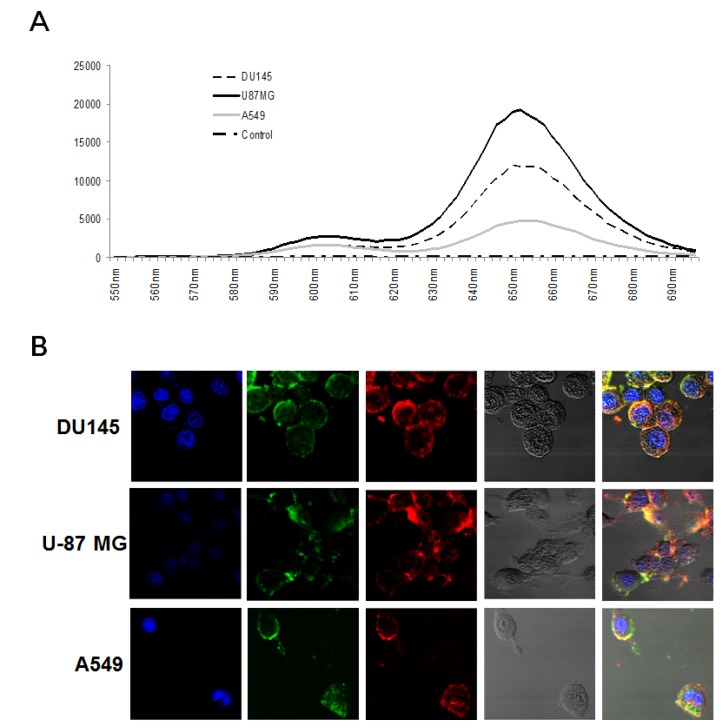
Simultaneous imaging of two cancer biomarkers in three different cancer cell lines. (**A**) Fluorescence emission spectra of a QD-TTA-1 and QD-AS1411 mixture in three cancer cell lines. DU145 (dotted black line), U-87 MG (solid black line), A549 (gray line), and baseline (dash-dotted line); excitation: 480 nm, scanning wavelength: 500–800 nm, with a bandwidth of 10 nm; (**B**) Confocal microscope images of cells incubated with two types of QD-aptamer conjugates. Column 1: DAPI (blue: 420–480 nm); column 2: QD-TTA-1 (green: 560–615 nm); column 3: QD-AS1411 (red: 647–690 nm); column 4: bright field; column 5: merged fluorescence image of DAPI and bright field and QD-aptamer conjugates.

Simultaneous imaging using a mixture of QD-TTA-1 and QD-AS1411 performed in each individual cell line and analyzed by confocal microscopy. The QD-aptamer conjugates mixture was stable in the three cancer cell lines. The fluorescence emission spectral shift was not detected in the cellular environment (Figure 5A). QD-TTA-1 and QD-AS1411 showed high fluorescence signals in the three cancer cell lines (Figure 5B). There were no significant differences among the three cancer cell lines. In all cancer cell lines, co-localization of QD-TTA-1 and QD-AS1411 was visualized as a yellow color because of the merging of green and red signals from each QD-aptamer conjugate. The nature of the interaction between tenascin-C and nucleolin in cancer, if any, is unknown. However, these results indicate that there may be an interaction or co-expression of the two proteins. Further studies using QD-aptamers could lead to new methods for studying interactions among or localizations of cancer-specific proteins.

Recent advances in nanotechnology, materials science, and surface chemistry have enabled the development of detection methods on the nano-scale. Semiconductor QDs are regarded as a new class of probes because of their small size ranges of 1–10 nm, which result in unique optical properties such as quantum confinement effects [15]. Their unique fluorescence emission spectra can be adjusted by controlling their chemical composition and size, and can be used to label materials for cellular imaging [16], fluorescent *in situ* hybridization (FISH) [17], and multiplexing [18]. Future cancer imaging technology demands high sensitivity and multiplexing capabilities, must be performed in real-time, and be high-throughput, label-free, and simple. Cancer-specific aptamer technologies should be incorporated into imaging technologies for use in clinical applications.

## 4. Conclusions/Outlook

In this study, two types of aptamer-conjugated QDs were developed and successfully applied for simultaneous imaging of two different cancer biomarkers using the specific targeting functionalities of aptamers. The imaging of tenascin-C using QD-TTA-1 provided a consistent and high fluorescence signal in three different cancer cell lines. Moreover, imaging of nucleolin using QD-AS1411 differed according to the type of cancer cell. These results demonstrated the cell line-dependent expression and distribution of cancer biomarkers. Thus, this method has potential applicability for intracellular localization or diagnosis of cancer biomarkers, and multiplex imaging of different cancer cell lines.
